# Multi-scale feature fusion network optimization and edge intelligent detection for transmission line insulator

**DOI:** 10.1371/journal.pone.0361403

**Published:** 2026-10-26

**Authors:** Chun Yang, Yining Ma

**Affiliations:** Energy Development Research Institute, China Southern Power Grid, Guangzhou, China; Swiss Federal Technology Institute of Lausanne, SWITZERLAND

## Abstract

In response to the accuracy loss caused by lightweight networks on edge devices and the difficulty in accurately detecting small defect targets, this paper studies the lightweight You Only Look Once version 8 (YOLOv8) transmission line insulator defect detection algorithm based on Three-Branch Attention Mechanism (TBAM) and Enhanced Multi-Scale Feature Fusion Network (EMSFFN), which can perform high-precision detection on edge intelligent devices of unmanned aerial vehicles. Aiming at the problem of significant differences in the number of image categories in the current aerial insulator defect dataset, the dataset was expanded through image data augmentation to generate more minority class samples, balance the number of samples in each category. Experiments show that compared with YOLOv7, YOLOv8, YOLOv9, Detection Transformer (DETR) and Faster Region-based Convolutional Neural Network (Faster R-CNN), the model parameters and computational complexity of TBAM-YOLOv8-EMSFFN are relatively low. Its AP reaches 99.2% and the mAP reaches 98.7%. Therefore, the TBAM-YOLOv8-EMSFFN model is suitable for unmanned aerial vehicles carrying edge intelligent devices to achieve power inspection.

## 1. Introduction

With the development and construction of smart grids, the scale of transmission lines is expanding rapidly [1]. To ensure the stable operation of the power grid system, it is necessary to carry out regular inspection work on transmission lines [2]. The main components of insulators on transmission lines are made of insulating materials such as ceramics and glass, ensuring electrical insulation between the current-carrying conductor and the ground [3]. Meanwhile, they can withstand the mechanical stress on the line and play a supporting role. Among various power grid accidents, safety incidents caused by insulator faults account for a large proportion [4]. Therefore, conducting regular and effective inspections and maintenance of insulators is an important task to ensure the stability and reliability of transmission lines.

Insulators play a role in preventing current leakage and maintaining effective isolation between each phase of the conductor and between the conductor and the ground on transmission lines [5]. Their performance depends on their good electrical insulation. However, as insulators are exposed to outdoor environments for a long time, they will be affected by various harsh conditions such as extreme cold and heat, alternating humidity and heat, wind and sand erosion, and air pollution, all of which may lead to the deterioration of the physical properties and reduced electrical performance of insulating materials, thereby increasing the risk of power failures [6]. Therefore, it is particularly important to adopt modern intelligent detection technologies and equipment, combined with traditional manual inspection methods, to carry out regular, comprehensive and precise detection and maintenance of insulators [7].

With the popularization of intelligent inspection by unmanned aerial vehicles, the application of artificial intelligence target detection technology to unmanned aerial vehicle inspection of transmission lines has become a trend [8]. In recent years, the gradual application of unmanned aerial vehicle inspection has made up for the deficiencies of traditional manual inspection in terms of cost and efficiency [9]. Due to the limited computing power at the edge of unmanned aerial vehicles, artificial intelligence target detection algorithms are rather complex [10]. Efficiently utilizing the limited space and computing power at the edge to compress the artificial intelligence target detection algorithms while ensuring high detection accuracy is of great significance.

This paper integrates Three-Branch Attention Mechanism(TBAM) into the feature map extraction network and the fusion network to enhance the extraction ability of defect feature information. A lightweight You Only Look Once version 8 (YOLOv8) that combines TBAM and Enhanced Multi-Scale Feature Fusion Network (EMSFFN) is proposed for insulator defect detection. To address the issue of unbalanced numbers of normal insulators and defect samples in the aerial insulator image dataset of transmission lines, various expansion datasets and image enhancement methods were adopted, thereby improving the situation where the aerial insulator image data was not rich enough and the training data was limited. Experiments show that TBAM-YOLOv8-EMSFFN significantly improves the detection precision of insulator defects in transmission lines.

The rest of this paper is organized as follows. Section 2 discusses the relevant work. Section 3 constructs the TBAM-YOLOv8-EMSFFN model. Section 4 presents the experimental results and conducts an analysis. Section 5 concludes the paper.

## 2. Related work

### 2.1. Research status of object detection based on deep learning

Deep learning simulates the working principle of the human brain and constructs a nonlinear variation neural network model to complete complex learning tasks [11]. Its main working principle is to extract and abstract features hierarchically. In the network, each layer can extract feature descriptions with different depths and levels and containing semantic information from the input data. With the continuous advancement of computer technology, the emergence of big data technology and cloud computing, as well as the further improvement of algorithms, deep learning has achieved rapid progress and significant breakthroughs in the field of object detection [12].

YOLOV7 inherits the excellent architecture of YOLOV5 and makes certain optimizations to it [13]. For instance, it introduces the Extended-ELAN module to balance the width and depth of the model, enabling it to perform better under different computing resources. Although single-stage networks have achieved considerable real-time performance and accuracy by now, in some platforms with relatively abundant computing power, two-stage object detection models still perform outstandingly [14]. At present, the more representative networks are the Region-based Convolutional Neural Network (R-CNN) series [15]. Compared with the direct forward propagation prediction of the YOLO series, the most significant feature of the two-stage network is that it uses an anchor box screening algorithm for screening before prediction. R-CNN conducts CNN feature extraction, and finally makes judgments through the judge and precisely locates them using the bounding box regression algorithm.

Fast R-CNN improves the algorithm process based on R-CNN. By proposing the idea of shared feature maps, it reduces repetitive calculations and improves the detection speed [16]. The joint training of candidate box generation and target detection is achieved by introducing the Region Proposal Network (RPN) structure. The subsequent emergence of Mask R-CNN, as a derivative of Faster R-CNN, expanded the application field of two-stage networks [17]. The subsequent networks proposed are all variant networks of Faster R-CNN proposed to address problems such as imbalanced samples of detectors and poor multi-scale detection capabilities in specific regions.

In summary, neither single-stage networks nor two-stage networks have absolute applicability or performance index advantages. The appropriate network model should be selected based on different scenarios and requirements, while ensuring real-time performance and taking into account a certain level of detection accuracy.

### 2.2. Research status of insulator fault detection

At present, the methods for detecting insulator defects in transmission lines are mainly divided into traditional methods and deep learning methods [18]. Traditional methods mainly rely on the shallow feature information of images, including texture analysis, edge detection, shape description, etc. They identify and locate abnormal areas of insulators through the extracted features and detect defects by constructing mathematical models [19]. For rotating insulators, relevant scholars have integrated the Canny edge extraction and the Speeded-Up Robust Feature (SURF) feature point detection algorithm, and used Harr wavelets to describe the feature points to achieve insulator identification [20]. Relevant scholars use the edge point algorithm to locally segment the insulator images of the catenary, and then automatically identify the insulator states based on their texture features [21].

Traditional methods rely on image processing, and their structures are designed manually, and they are easily disturbed by external conditions such as light and temperature. It does not have the characteristic of strong universality for faults under different working conditions, and there are often significant limitations in engineering applications. Some researchers have begun to apply advanced target detection algorithms to the research on insulator detection [22]. Relevant scholars have proposed an insulator target recognition scheme that selects DarkNet and ResNet as the core network modules [23]. It is concluded that the detection effect of the DarkNet as the backbone network is better than that of the ResNet as the backbone network. Relevant scholars have proposed a fast detection method for insulator string images optimized based on the YOLOv3 algorithm [24]. Based on the original YOLOv3 network framework, targeted simplification processing was carried out, and the prior boxes suitable for insulator string target detection were screened out by using the clustering method. This measure effectively improved the real-time response speed and effect of insulator detection. Relevant scholars have proposed an insulator low-zero value fault diagnosis method that integrates the HSV color space characteristics and YOLOV3 target detection technology [25]. The HSV color model is used to perform spatial feature normalization correction processing on the image. By combining the intrinsic correlation between each component of HSV and temperature, the fault state of insulators is determined.

Although there are many studies on insulator fault detection utilizing deep learning and many achievements have been made, there are few studies on multi-scale feature networks for insulator fault detection. Most studies focus solely on the detection of single-mode insulators. Due to the complex fault environment and types of insulators, advanced intelligent models are needed to detect their operating status.

## 3. Methodology

### 3.1. Integration of TBAM

The attention mechanism enables models to capture more valuable information and focus on the desired target area. TBAM is incorporated into the core feature extraction network. With limited parameters, the network has the ability to extract more crucial information from the input images. The spatial attention mechanism is dedicated to learning and enhancing the pixel information of spatial positions, focusing on determining key position information. TBAM represents the integration of two technologies. It requires the consolidation of attention in space and channels, the fusion of multi-dimensional information exchange, the integration of information in channels and space, and the operation with fewer parameters.

Fig 1 shows the specific structure of TBAM. TBAM adopts an architecture where three branches operate synchronously. The first two branches respectively complete cross-channel information interaction along the height and width dimensions, while the remaining third branch is responsible for learning spatial attention. The Z-Pool operation first combines the results of average pooling and maximum pooling, and then outputs the final result through average aggregation. With only a small increase in parameter cost, channel attention and spatial attention can be integrated together.

**Fig 1:**
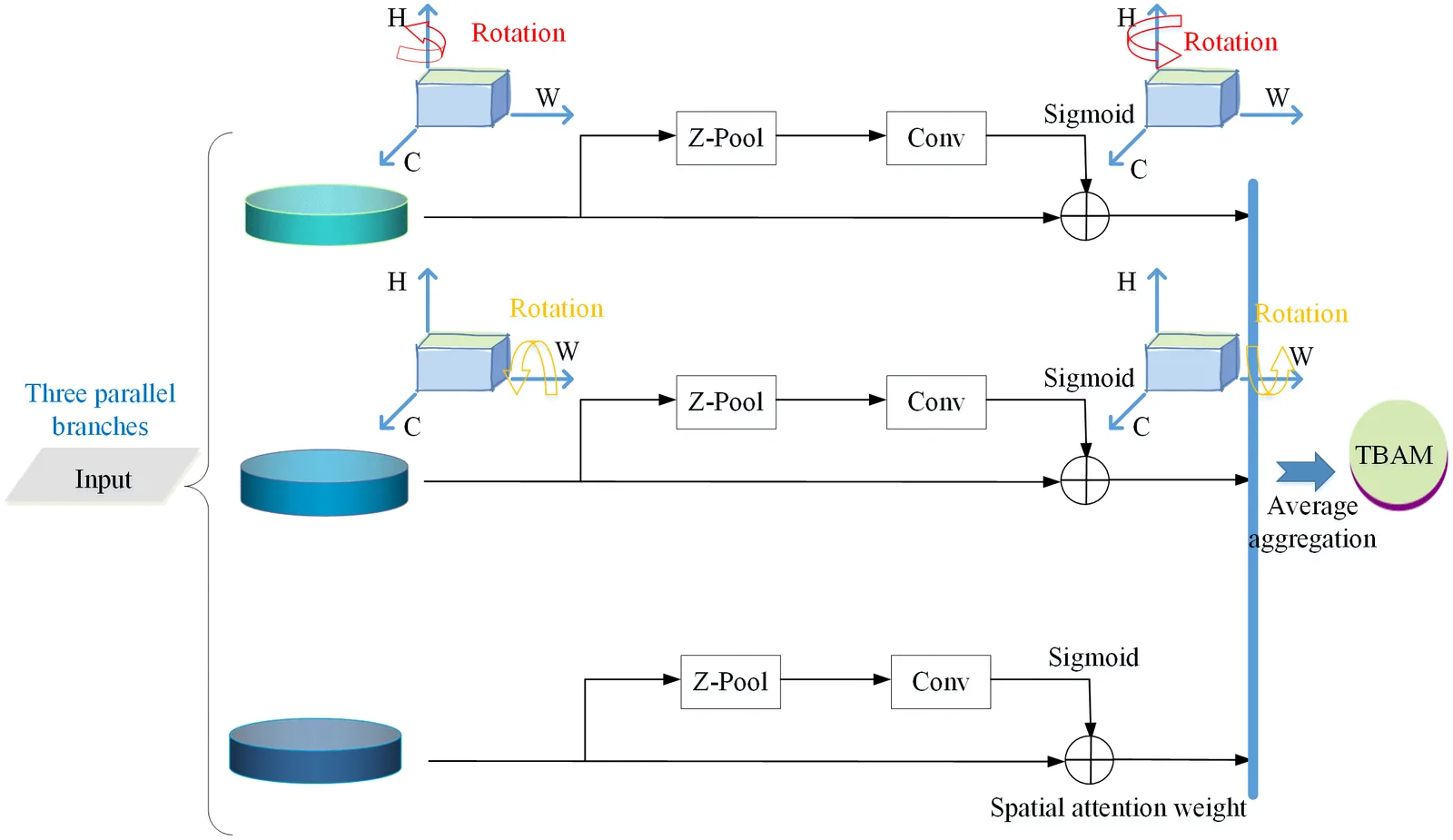
TBAM structure.

Suppose the input is *X ∈ R*^*C×H×W*^, where *C* is the number of channels, *H* is the height, and *W* is the width. When passing through the first branch, the tensor is rotated along the height dimension *H*, expressed as *X*_*1*_ *∈ R*^*W×H×C*^. The generated attention weights need to be rotated again to maintain dimension consistency with the input tensor. Similarly, when passing through the second branch, the tensor rotates along the width dimension *W*, expressed as *X*_*2*_ *∈ R*^*H×C×W*^. Through similar operations, the dimensions of the output tensor are kept consistent with those of the input tensor. For the third branch, the channel *C* of the input tensor directly passes through the *Z*-Pool operation to obtain *X*_*3*_ *∈ R*^*2×H×W*^. Finally, the final output tensor is obtained by averaging and aggregating the three branches. Specifically, the *Z*-Pool operation reduces this dimension to 2 by concatenating the average pooling and maximum pooling along the 0th dimension of the tensor. The *Z*-Pool operation is shown in the following formula, where *d0* represents the 0th dimension.


$$\begin{array}{c} Z-Pool\left(X\right)=\left[MaxPool_{d0}\left(X\right),AvgPool_{d0}\left(X\right)\right] \end{array}$$ (1)

### 3.2. YOLOv8 detection algorithm

In the C2f module of YOLOv8, the input is processed by the CBS convolution module and then the Split operation divides the feature map with *C* channels output by the CBS module into two feature maps with C/2 channels, thereby reducing the computational load and memory consumption. The internal Network structure diagram of C2f and the structure of Path Aggregation Network (PAN) +Feature Pyramid Network (FPN) are shown in Fig 2.

**Fig 2:**
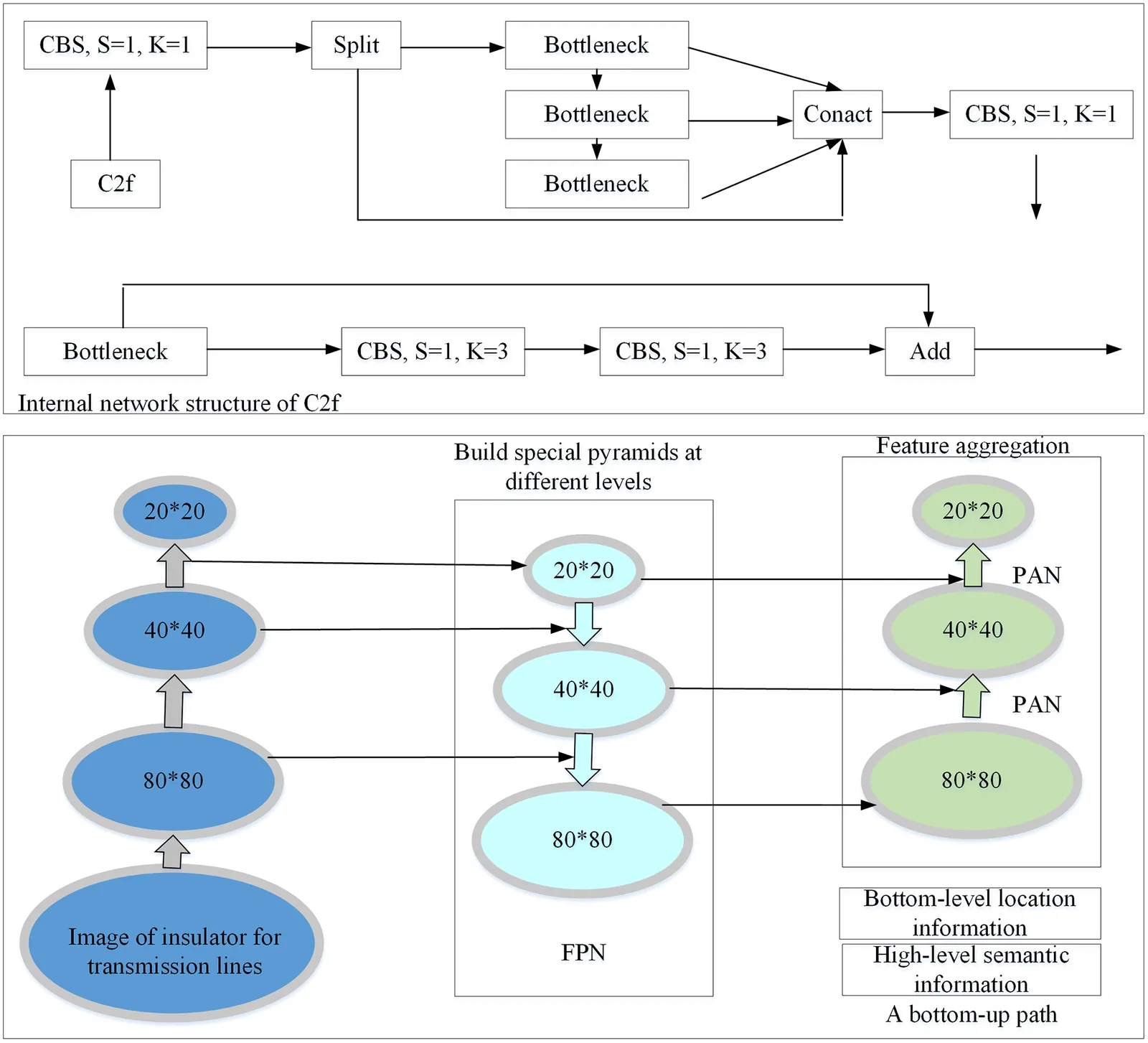
Internal network structure diagram of C2f and the PAN + FPN structure.

The neck design of YOLOv8 adopts the combined design concept of PAN + FPN. FPN constructs feature pyramids at multiple levels, allowing the model to detect objects at various scales, while PAN is used to integrate these features. PAN adopts a bottom-up strategy, transmitting low-level feature information step by step to high-level and integrating it with corresponding FPN features. This design scheme enables the model to more effectively utilize low-level positional information and high-level semantic information. In addition, PAN aggregates features at different levels through horizontal connections, further enhancing the expressive power of features. After processing by the Neck layer, the network can output various levels of features.

The head region is responsible for the final target classification of various levels of features and conducting regression analysis of bounding boxes. Varifocal Loss (VFL) is adopted as a function of classification loss. Regression loss is a combination of Complete Intersection over Union (CIoU) loss and Distribution Focal Loss(DFL). The CIoU loss is actually an optimized version of the bounding box regression loss function, which comprehensively considers three major factors: the overlapping area between the predicted box and the actual box, the relative distance between the center point, and the ratio of its longitudinal and transverse directions. Compared to traditional IoU loss, CIoU loss can more deeply evaluate the similarity between predicted boxes and real boxes, thereby providing more accurate bounding box prediction guidance for the model. The mathematical formula for CIoU is as follows:


$$\begin{array}{c} CIoU\_Loss=1-IoU+p^{\text{2}}\left(b,b^{gt}\right)c^{-2}+\alpha v \end{array}$$ (2)

$$\begin{array}{c} v=4\pi^{-2}{\left[arctan\left(w^{gt}h^{-gt}\right)-arctan\left(wh^{-1}\right)\right]}^{\text{2}} \end{array}$$ (3)

$$\begin{array}{c} \alpha =v{\left(1-IoU+v\right)}^{-1} \end{array}$$ (4)

*c* indicates the diagonal distance that can simultaneously cover the minimum closed area of both the predicted box and the real box. *b* and *b*^*gt*^ respectively represent the center points of the prediction box and the real box, *p* represents the Euclidean distance between the two center points, *α* represents the weight function, *v* is used to measure the similarity of the aspect ratio, *w*^*gt*^*/h*^*gt*^ represents the aspect ratio of the real target box, and *w/ℎ* represents the aspect ratio of the prediction box.

The traditional coordinate point regression technique adopts the Dirac distribution, which is based on the assumption that the labels are absolutely accurate. However, in practical application scenarios, the boundaries of the target box exhibit a certain degree of uncertainty when occluded or blurred. Traditional regression techniques cannot effectively deal with this uncertain problem. To address this issue, DFL adopts a modeling method for the probability distribution of bounding box positions, as shown in the following formula, where *S* represents the output value of the probability.


$$\begin{array}{c} DFL\left(S_{i},S_{i+1}\right)=-\left[(y_{i+1}-ymathrmlog\left(S_{i}\right)+(y-y_{i})log\left(S_{i+1}\right)\right] \end{array}$$ (5)

The VFL loss function is an advanced version of the Focal loss function, used to train object detectors to predict IoU-aware classification scores. The calculation formula for VFL is as follows:


$$\begin{array}{c} VFL\left(p,q\right)=\left\{ \begin{array}{cc} @cc@-q\left[qlog\left(p\right)+(1-qmathrmlog(1-p)\right] & q>0\\ -\alpha p^{\gamma }log(1-p) & q=0 \end{array}\right. \end{array}$$ (6)

Among them, *q* is the target IoU score, *p* is the predicted IoU-aware classification score, *α* is used to balance the weights of positive and negative samples, and *p*^*Υ*^ is used to modulate the weights of each sample.

### 3.3. Optimization of the multi-scale feature fusion network

When conducting defect detection on transmission line insulators, identifying small-scale and high-density targets is particularly challenging. This is because their representation range on the feature map is relatively small. Therefore, the network needs to have fine feature extraction and fusion capabilities.

Feature fusion networks can efficiently fuse backbone features, extract features at all levels from the network, and integrate surface nuances with deep semantic abstract information. This fusion method can retain the information of the image to the greatest extent before the classification and localization stages of object detection, thereby reducing the information loss that may be caused by continuous downsampling.

In the object recognition task based on deep learning, integrating features of various scales can effectively enhance the performance of the model. Deep networks have a broader receiving range and can capture a large amount of semantic data. However, due to their low resolution, the information they provide is not very detailed. In comparison, although shallow networks perform better in terms of resolution and geometric detail representation, their ability to capture semantic information is relatively weak. Therefore, in the process of optimizing the object detection algorithm, effectively integrating the advantages of these two characteristics is particularly crucial.

Due to the difference in photographic distance, the defect targets of insulators in aerial images occupy a relatively small portion of the overall image, especially in complex background environments. However, the downsampling factor of YOLOv8 is relatively high and cannot meet the requirements for learning the small target features of insulator defects, which has a negative impact on the detection effect.

Fig 3 shows the enhanced multi-scale feature fusion network. Based on the original Path Aggregation Network (PANet), two layers of shallow feature maps (160 × 160 and 320 × 320) were added. After detecting the feature map of 160 × 160 scale, the classification prediction network was added to the network output end. The *C*2 (160 × 160) and *C*1 (320 × 320) feature maps output by the backbone network were introduced into the FPN structure. In this process, *M*3 (80 × 80) is upsampled and then concatenated with *C*2, and then processed by the C2f module to generate *M*2 (160 × 160). Subsequently, *M*2 was also upsampled and concatenated with *C*1 to generate *M*1 (320 × 320). In terms of PAN path, *P*1 (320 × 320) performs downsampling operation first, and then merges with *M*2 to generate a new *P*2 (160 × 160). After *P*2 in the network, a prediction head Head4 is also added for detecting small targets, and a TBAM module is introduced before this prediction head. EMSFFN not only enhances the perception ability of small targets with insulator defects in this way, but also has good performance in computational efficiency.

**Fig 3:**
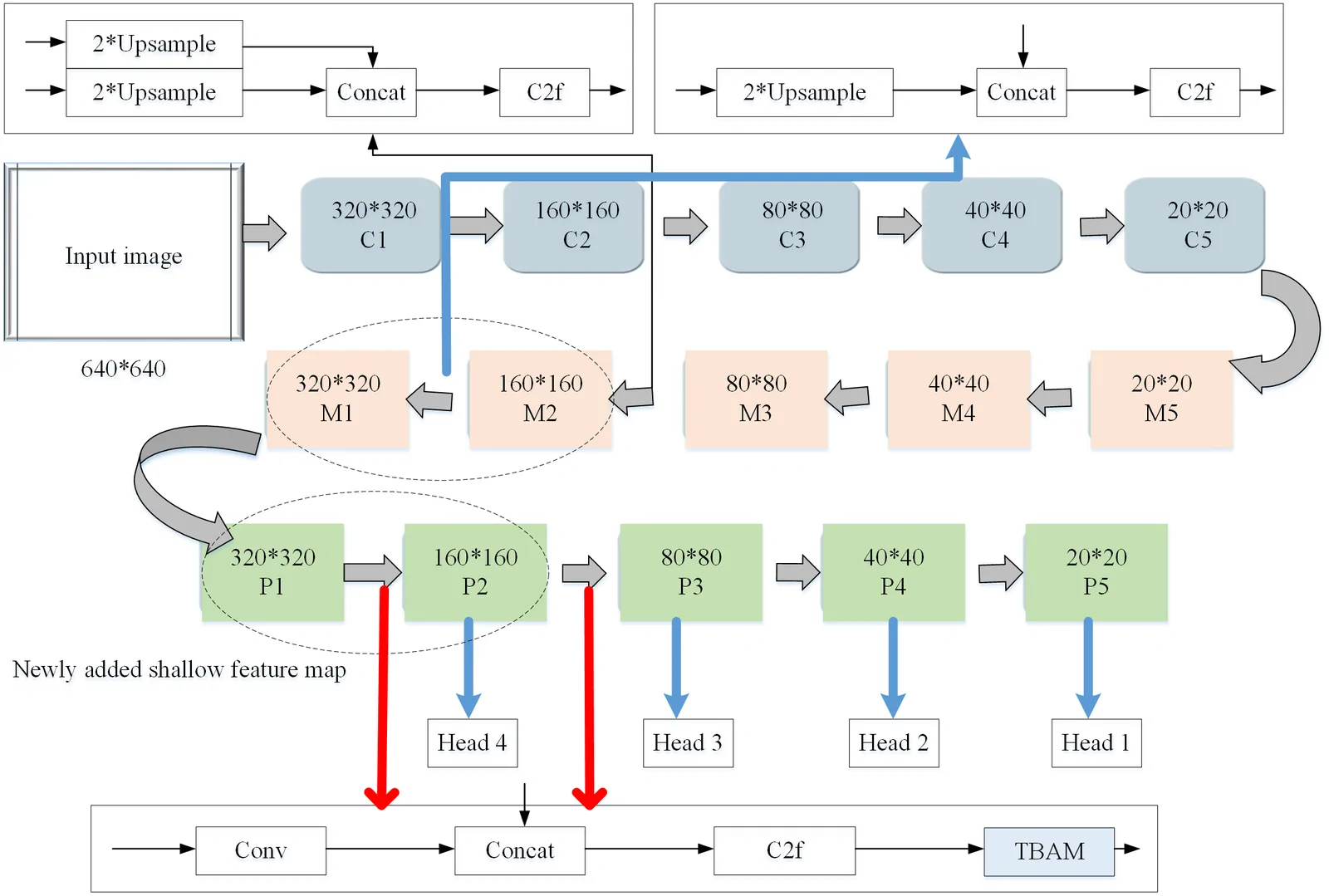
Enhanced multi-scale feature fusion network.

The implementation path of EMSFFN is different from that of conventional FPN, PAN, and BiFPN. It uses YOLOv8 as the existing base, adds 160 × 160 and 320 × 320 shallow feature maps, and adds a specialized prediction head for small targets at the end of the 160 × 160 feature map. The pre-module connected to the prediction head is replaced with TBAM. This architecture adjustment can enhance the model’s ability to recognize small-scale insulator defects, and the resource occupation during the operation process can also be maintained at a relatively low level.

The batch of feature maps is named the *C* series. *C*1 to *C*5 represent the feature map mappings of different resolutions obtained by downsampling the original feature map at different multiples. The feature maps of the *C* series are input into the FPN network. Features are passed from top to bottom through upsampling and feature joining, thereby generating the feature maps of the *M* series. More specifically, the *M*3 (80 × 80) feature map was connected with the *C*2 (160 × 160) feature map using the double upsampling method, and the newly added *M*2 (160 × 160) feature map was obtained through the C2f convolution module.

The feature maps of the *M* series are input into the PAN network, and then feature transfer is carried out from bottom to top through downsampling and feature cascading, thereby generating the predicted feature maps of the *P* series.

Constructing an enhanced multi-scale feature fusion network for targets with smaller defects can further promote the interaction between adjacent features, fully explore and utilize the extracted multi-scale features. In order to better improve the network’s recognition ability for small defect targets, a shallow feature map (*P*2) with a scale of 160 × 160 is generated in the enhanced multi-scale feature fusion network. At the corresponding network output end, a prediction head has been added, mainly for the detection of small targets. This feature map shows a relatively small perceptual range and relatively rich local detail information. By adding a TBAM module before the prediction head to update the previous feature map, the network’s ability to extract key features has been enhanced, which helps to more effectively integrate shallow graphic features and deep semantic features.

Compared with existing methods, the EMSFFN proposed in this paper introduces two shallow feature maps with sizes of 160 × 160 and 320 × 320 for the first time on the basis of YOLOv8, and also adds a prediction head for small targets to solve the shortcomings of lightweight networks in perceiving small targets. TBAM adopts a three branch structure to efficiently integrate channel and spatial attention, thereby compensating for the shortcomings of methods like SE. Combining EMSFFN and TBAM can achieve an ideal balance between accuracy and efficiency in application scenarios such as edge intelligence.

### 3.4. Improved network structure

To solve the problem of difficulty in accurately identifying small-sized defect targets in complex backgrounds, TBAM is integrated into the backbone feature extraction network and the feature fusion network to suppress useless information such as complex backgrounds, making the network more focused on key areas and thereby improving the accuracy of defect detection. In addition, the feature fusion network was improved. An enhanced multi-scale feature fusion network was designed. Two layers of shallow feature maps with scales of 160 × 160 and 320 × 320 were introduced, and a new classification prediction network was added after the 160 × 160-sized feature map.

The optimized insulator defect detection method TBAM-YOLOv8-EMSFFN mainly consists of three core parts: the YOLOv8 feature extraction network, the enhanced multi-scale feature integration network, and four prediction heads, as shown in Fig 4.

**Fig 4:**
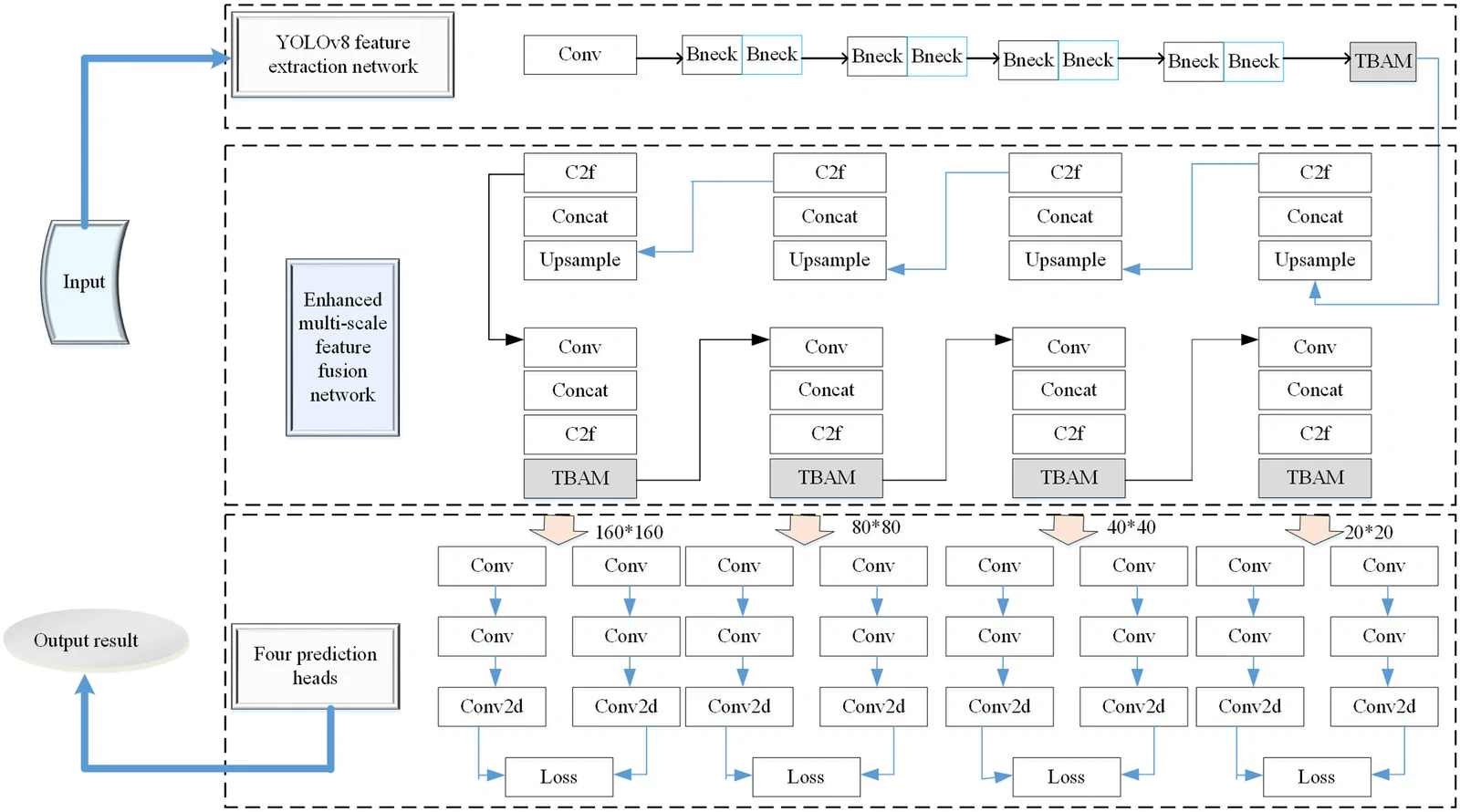
Network structure of TBAM-YOLOv8-EMSFFN.

In view of the limitations of the CIoU loss function in the insulator defect detection task, a specialized improvement was made to it, and a loss function EDIoU based on endpoint distance was proposed.

The principle formula of EDIoU is shown in Equations (7), (8), and (9):


$$\begin{array}{c} L_{EDIoU}=1-IoU+\left(c_{\text{1}}^{\text{2}}+c_{\text{2}}^{\text{2}}\right){\left(w^{\text{2}}+h^{\text{2}}\right)}^{-1} \end{array}$$ (7)

$$\begin{array}{c} c_{\text{1}}^{\text{2}}={\left(x_{\text{1}}^{gt}-x_{\text{1}}\right)}^{\text{2}}+{\left(y_{\text{1}}^{gt}-y_{\text{1}}\right)}^{\text{2}} \end{array}$$ (8)

$$\begin{array}{c} c_{\text{2}}^{\text{2}}={\left(x_{\text{2}}^{gt}-x_{\text{2}}\right)}^{\text{2}}+{\left(y_{\text{2}}^{gt}-y_{\text{2}}\right)}^{\text{2}} \end{array}$$ (9)

In the formula, *c*_*1*_ represents the distance between the upper left endpoint of the prediction box and the real box. *c*_*2*_ is the distance between the lower right endpoint of the prediction box and the real box. *w* and *h* are respectively the length and width of the prediction box.

In the improved loss function, penalty terms for two endpoints are introduced. The aspect ratio penalty term added to the CIoU and EIoU loss functions has been simplified. The improved EDIoU comprehensively considers the relevant factors in the existing loss function, and optimizes during the calculation process, thereby better capturing the relative positional relationship between bounding boxes and enhancing the sensitivity to the details of defect edges.

In this experiment, under the condition of completely unified training process and test parameters, two loss functions, EDIoU and CIoU, were compared. EDIoU set separate penalty terms for the top-left corner point and the bottom-right corner point of the predicted box, simplifying the original penalty part based on the aspect ratio, and the resulting loss values can more accurately capture the position offset of the defect edge. The comparison data of all groups were uniformly organized and placed in Table 1.

**Table 1:** Comparison of CIoU and EDIoU.

Loss function	AP(%)	mAP(%)	Localization error (pixels)	Convergence epoch
CIoU	98.6	98.1	2.8	180
EDIoU	99.2	98.7	2.1	160

As shown in Table 1, there are clear differences in the performance indicators between EDIoU and CIoU. The average precision of EDIoU has increased by 0.6 percentage points, the positioning error has decreased by 0.7 pixels, and the number of rounds required for convergence has been reduced from the original 180 rounds to 160 rounds. EDIoU can provide more accurate positioning references at the edge positions of insulator defects, and is more adaptable in scenarios of small target recognition and fuzzy defect detection.

## 4. Experimental results and analysis

### 4.1. Dataset construction and preprocessing

Building and preprocessing datasets is a crucial step in model training. The quality of the dataset directly affects the performance and generalization ability of the model.

The intelligent detection algorithm for multiple types of transmission line insulator defects was trained using the insulator dataset collected by the Energy Development Research Institute of China Southern Power Grid Co., LTD. This dataset uses unmanned aerial vehicles to capture and collect aerial images of insulators and their defects on transmission lines. The sample background includes fields, mountains and forests, skies, buildings, etc. The background and defect types are rich, which meets the actual needs of insulator inspection for transmission lines.

There are 3,200 images in the original data set, and there is imbalance among the categories, including about 2,100 images of normal insulators, 400 images of self-explosion defects, 350 images of breakage defects and 350 images of flashover defects respectively.

In order to alleviate the imbalance of categories and enhance the generalization ability of the model, geometric transformation, random rotation and adding noise are used to expand the defect samples. After such processing, the obtained data set contains a total of 6,400 pictures, including 2,100 normal insulators, 1,500 self-explosion defects, 1,400 breakage defects and 1,400 flashover defects.

When dealing with images from deep learning-based object detection datasets, it is necessary to create an image annotation file. This study selected LabelImg as the tool for image annotation. This software is developed based on the Python language and is a commonly used visual image annotation tool. It supports multiple annotation formats. The YOLO format is adopted to directly generate the labels required for training the YOLO object detection algorithm. In this paper, label 0 indicates normal insulator, label 1 indicates self-explosion defect insulator, label 2 indicates damaged defect insulator, and label 3 indicates flashover defect insulator.

In order to prevent cross leakage of data, this paper performs subset segmentation on all original images. 3200 original images are divided into training set, verification set and test set according to the ratio of 8:1:1. In the segmentation process, the two dimensions of shooting time and transmission line are combined for hierarchical processing. Geometric transformation, random rotation and noise enhancement operations only work on the training set, and produce more samples belonging to a few categories. All contents in the verification set and test set directly use the original image without any enhancement operation. Enhanced versions derived from the same original image are grouped into the same subset. No enhanced image will be included in the verification set or test set. Five-fold cross-validation was performed within the training set, and transmission line samples not previously involved in the training process were selected as an independent test set to evaluate the model’s generalization ability.

Five-fold cross-validation operation was conducted on the training dataset. Each division would split the complete training set into five independent subsets. Four of these subsets would complete the training process, while the remaining one would be used for the validation operation. Throughout the process, the content of the common test set and the unseen line test set remained unchanged, and no adjustments were made to the contents of the two data sets. When conducting the validation related to unseen lines, the transmission line number was set as a grouping variable to ensure that all images corresponding to the same line would only be assigned to the same subset.

### 4.2. Experimental environment and training parameters

The Windows 10 operating system is taken as the platform for the experimental research. The hardware configuration is a GeForce RTX 3060 graphics card, a video memory size of 6GB, and 32GB DDR5 memory. Software environments were set up using python3.8, pytorch1.11.0, torchvision0.12.0, and cudatoolkit11.3 through Anaconda. The hyperparameter settings for model training are shown in Table 2.

**Table 2:** Hyperparameter settings for model training.

Name	Parameter	Name	Parameter
Input size of the image	640 × 640	Total number of training rounds	200
Stop early and wait for the number of turns	100	Learning rate momentum	0.937
Number of threads	8	Initial learning rate	0.05
Batch size	16	Final learning rate	0.01

Among them, the number of early stop waiting rounds is a new hyperparameter added by YOLOv8, which is used to determine the number of waiting rounds. If no significant improvement in model performance is observed after exceeding this number of rounds, the training is stopped. The purpose of early stop is to reduce overfitting. Overfitting refers to the situation where a model can only fit the training data but cannot well fit the states of other data not included in the training data. By stopping early, it is possible to prevent performance degradation on validation data when the model performs overly well on training data.

### 4.3. Comparative experiments on attention mechanisms

To ensure the accuracy of the experiments, two different experiments on the attention mechanism were conducted in this section: one was a comparison experiment of the fusion positions, and the other was a contrast experiment of the fusion of different attention mechanisms.

TBAM was fused at various positions within the YOLOv8-EMSFFN network, as shown in Fig 5. Group 1 adopted the YOLOv8-EMSFFN model without TBAM fusion. Group 2 incorporated TBAM fusion only at the end of the backbone network. Group 3 only added TBAM to the feature fusion network. Group 4 added TBAM at two different positions.

**Fig 5:**
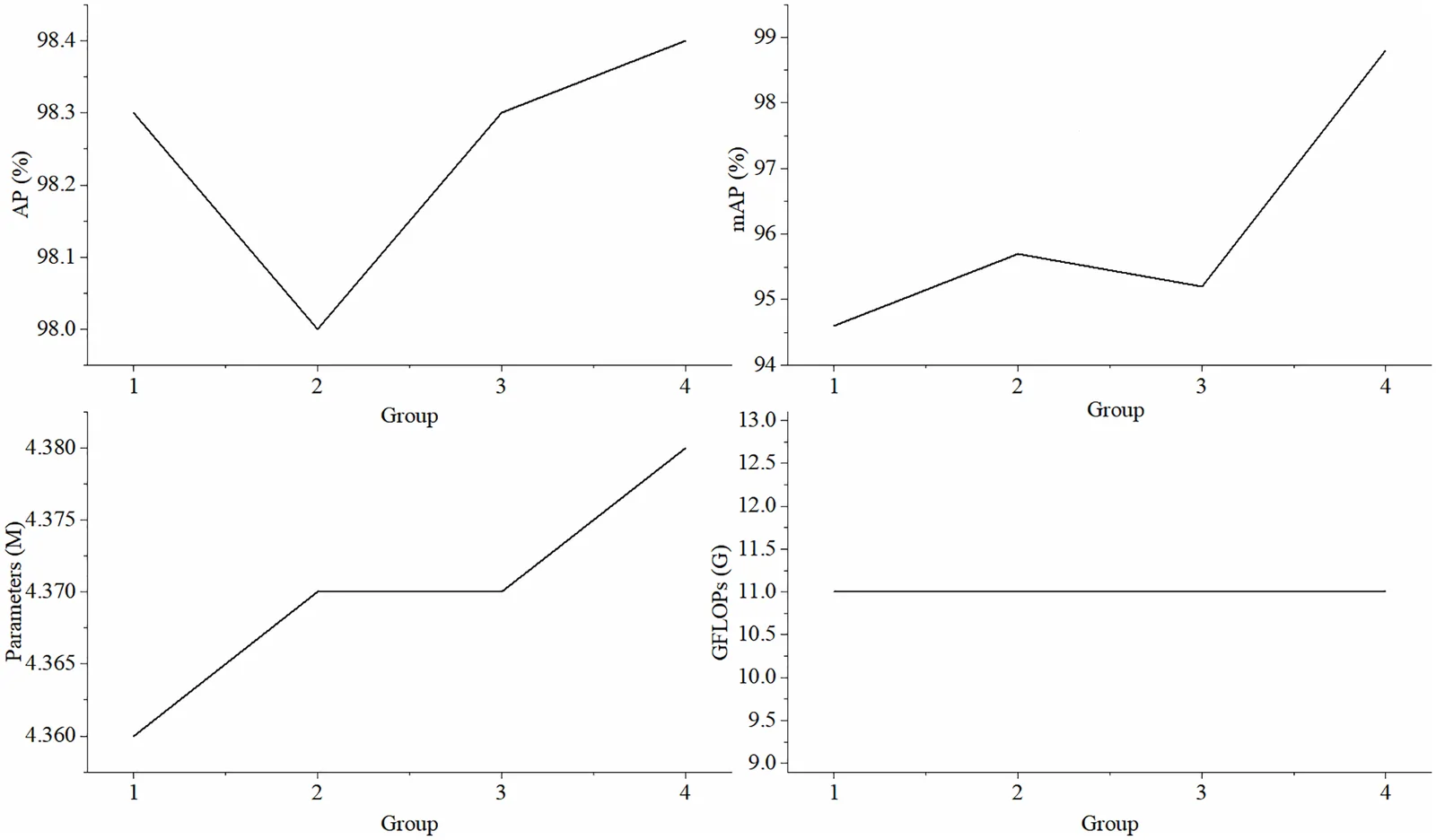
Comparison experiment of TBAM fusion position.

As shown in Fig 5, adding TBAM has a relatively small impact on the number of parameters and computational load of the network, especially in terms of computational load, there is no significant change.

When TBAM is added at both positions, the model performance is the best, and the detection accuracy of small defect targets is significantly improved. This indicates that TBAM can better focus on the features of the key defect areas, thereby accurately detecting defects. When added only at the end of the backbone network, the performance is also significantly improved, effectively enhancing the utilization rate of feature extraction, with a mAP of 95.7%. When added to the feature fusion network, the performance slightly improves, but it is relatively low, with a mAP of 95.2%. The model without TBAM integration has the lowest performance, with a mAP of only 94.6%.

To study the influence of the fusion of different attention mechanisms on the recognition accuracy of the model, under the premise of maintaining the same hyperparameters, the fourth group was selected, that is, attention mechanisms were added at two different positions. To verify the practicality of TBAM, comparative experiments were conducted using Squeeze-and-Excitation (SE), Efficient Channel Attention (ECA), and Convolutional Block Attention Module (CBAM). In the horizontal coordinate of Fig 6, groups 1–5 respectively represent the mechanisms of no attention, SE, ECA, CBAM and TBAM.

**Fig 6:**
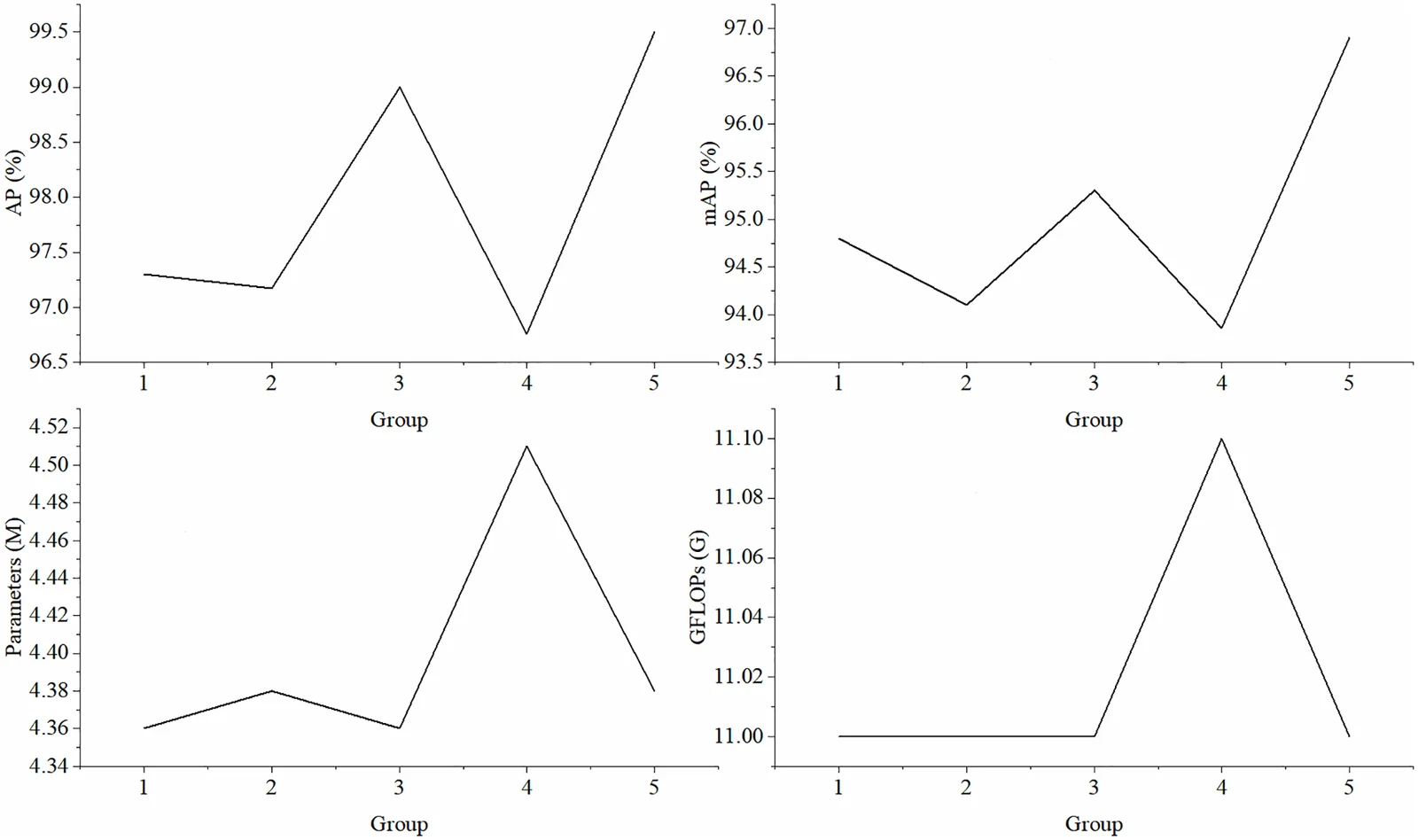
Comparative experiments of different attention mechanisms.

From the experimental data shown in Fig 6, the growth of TBAM was the most significant, mAP increased by 2.1 percentage points, and AP also rose by 2.2 percentage points. In addition, TBAM can obtain relevant information of a wider area in the network without adding additional computing power. This significantly enhances the network’s emphasis on object detection, thereby optimizing the overall performance.

However, after integrating the SE and CBAM attention mechanisms into YOLOv8-EMSFFN, the detection performance of the network decreased rather than increased. Both the defect detection accuracy and the average detection accuracy were lost, among which the CBAM accuracy loss was the greatest and the increased complexity was also the greatest. This indicates that after the addition of SE and CBAM, the channels and position information of the input features were not effectively mined, and the effective information of the deeply dependent features was not obtained, resulting in a decrease in the accuracy of the object detection network.

Referring to the test results presented in Fig 6, after connecting the SE and CBAM modules to the model, the overall performance of the target detection declined. The accuracy loss brought by the CBAM module was the greatest among all the modules, and its corresponding computational overhead was also at the highest level. The reason for this situation is that the CBAM module, in a sequential manner, calls the channel attention branch and the spatial attention branch to complete the calculation. In the shallow feature layers of the network, small defect targets with a very low proportion of pixel volume are easily filtered out by the spatial attention branch, and the original weak defect feature feedback is not provided.

The design of the SE module only models the dependency relationship in the channel dimension and does not set corresponding settings for the positioning function related to spatial positions. The TBAM module adopts a parallel structure of three branches combined with the Z-Pool operation, which can retain all information in the channel dimension and spatial dimension during the processing. During the operation of the basic benchmark model, it is interfered by irrelevant backgrounds. The CBAM module filters out some features that belong to the defects, while the TBAM module can precisely extract features in the area where the defect is located.

### 4.4. Ablation experiment

To visually demonstrate the individual roles of the TBAM module and the EMSFFN module, an ablation comparison experiment at the component level was conducted. Based on the statistical results in Table 3, it can be observed that when the TBAM module is embedded at the end of the backbone network, the average accuracy of the model increases from the original 94.6% to 95.7%. When the TBAM module is embedded in the feature fusion network section, the average accuracy of the model rises to 95.2%. When both the specified positions are embedded with the TBAM module, the average accuracy of the model can reach 98.7%. The combination of these two modules can form a positive synergy effect.

**Table 3:** Component-level ablation of TBAM and EMSFFN.

Group	TBAM in Backbone	TBAM in fusion‑network	160 × 160 feature map	160 × 160 prediction head	320 × 320 prediction head	AP(%)	mAP(%)	Parameters(M)
1	–	–	–	–	–	98.3	94.6	4.36
2	Yes	–	–	–	–	98.0	95.7	4.37
3	–	Yes	–	–	–	98.3	95.2	4.37
4	Yes	Yes	–	–	–	98.4	98.8	4.38
5	Yes	Yes	Yes	Yes	–	99.2	98.7	4.12
6	Yes	Yes	Yes	Yes	Yes	98.7	95.9	4.41

For the testing section of the EMSFFN module, after connecting the shallow feature map with a size of 160 × 160 and the corresponding prediction head, the model’s detection ability for small target defects significantly improves, and the final model parameters amount to 4.12M, with a computational cost of 17.7G, achieving an average accuracy of 98.7%. Connecting a prediction head with a size of 320 × 320 doubles the overall computational cost of the model, but the average accuracy drops to 95.9%.

Excessive shallow feature information mixed together can cause conflicts at the feature level, and the final version of the model only retains the prediction head with a size of 160 × 160. The differences in average accuracy between the 4th experimental group and the 5th experimental group are less than 0.1 percentage points, which is within the normal fluctuation range during the experiment.

To make the statistical results reliable, all experimental groups uniformly apply the completely consistent data division rules, set the same super parameters and adopt the completely same training process. Each group ran independently for five times to repeat the test, and finally calculated the average precision mean and corresponding standard deviation of all results. The consideration of setting five repeated tests is to strike an appropriate balance between the stability of statistical results and the overall calculation cost. Using paired t-test method, the benchmark model and the improved model are evaluated on the same test set and the same partition, which can offset the variable interference caused by the data partition stage and random initialization. The average accuracy of TBAM-YOLOv8-EMSFFN is 98.7%, and the corresponding standard deviation is 0.12%. The average accuracy of the benchmark model YOLOv8-EMSFFN is 94.6%, and the corresponding standard deviation is 0.23%. The p value calculated by paired t-test was lower than 0.01, and there was a significant difference between the two groups. During the research process, the effect quantity is calculated synchronously to measure the actual extent of performance improvement.

### 4.5. Cross-validation and unseen-line generalization experiments

The average precision values obtained from the five experiments were 98.6%, 98.8%, 98.7%, 98.5%, and 98.9% respectively, as shown in Table 4. The performance of the model varied very little under different data splitting schemes. Using new transmission line data that had not participated in the training for testing, the average precision value obtained was 98.5%, the mean average precision value was 97.8%, and the recall value was 97.6%. This model can cover the sample data of transmission lines that have not been encountered before.

**Table 4:** Results of five-fold cross-validation and unseen-line validation.

Validation scheme	Fold	AP(%)	mAP(%)	Recall(%)
Five-fold cross-validation	1	99.1	98.6	98.5
Five-fold cross-validation	2	99.3	98.8	98.7
Five-fold cross-validation	3	99.2	98.7	98.6
Five-fold cross-validation	4	99.0	98.5	98.4
Five-fold cross-validation	5	99.4	98.9	98.8
Unseen‑line test	–	98.5	97.8	97.6

### 4.6. Comparative experiment of object detection networks

To verify the advantages of the TBAM-YOLOv8-EMSFFN network over other object detection algorithms, network models of different object detection algorithms were trained on the transmission line insulator dataset. The evaluation indicators of the six network models are shown in Fig 7. The labels 1–6 on the horizontal axis represent YOLOv7, YOLOv8, YOLOv9, Detection Transformer (DETR), Faster R-CNN and TBAM-YOLOv8-EMSFFN respectively.

**Fig 7:**
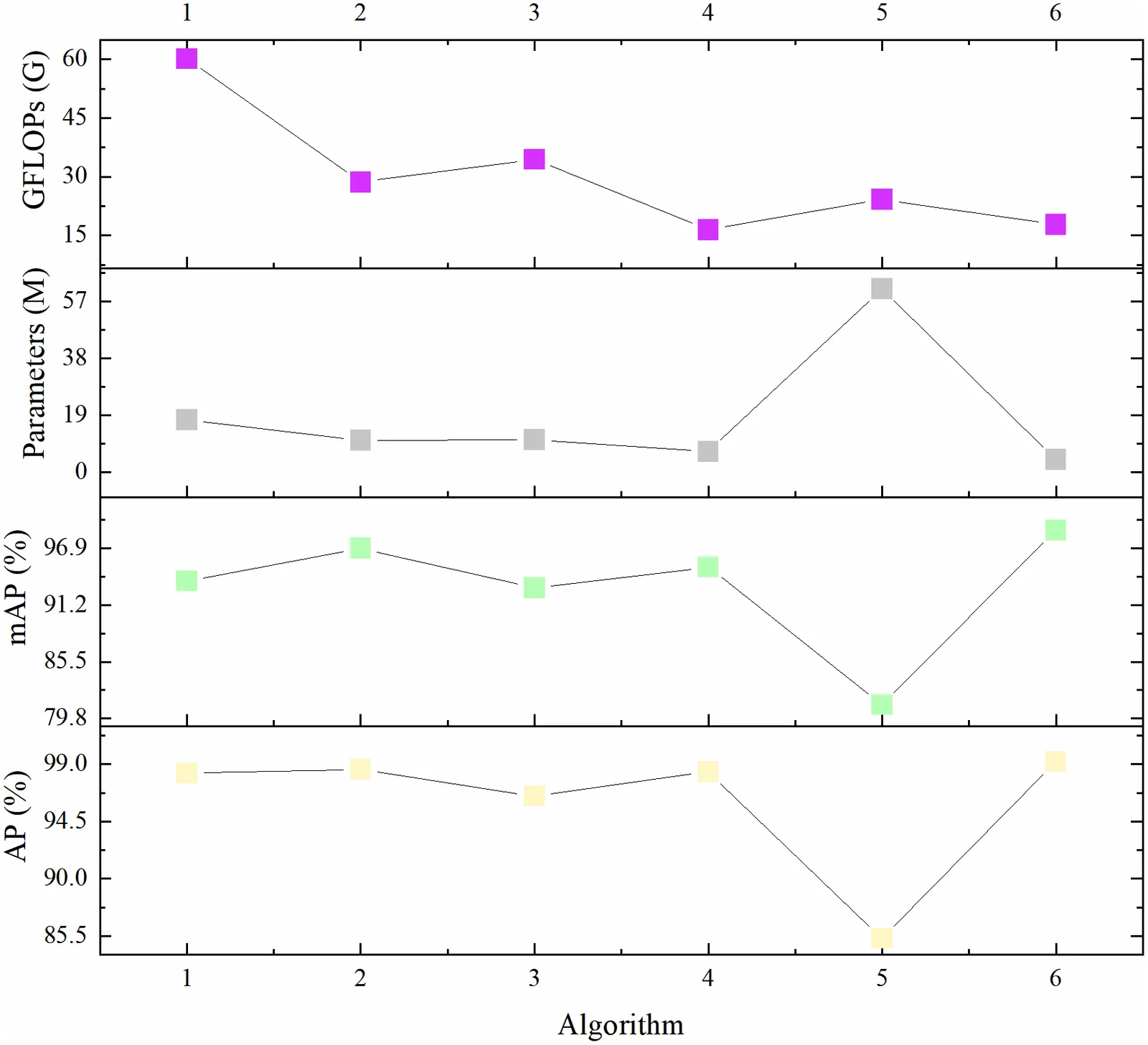
Comparative experiment of the object detection network.

Based on the detection data of the six network models shown in Fig 7, TBAM-YOLOv8-EMSFFN demonstrates more advantageous performance in terms of AP, mAP, Parameters and GFLOPs in insulator defect detection compared with the other five models. In comparison, other detection methods are relatively insufficient in generalization ability, which leads to low precision in insulator defect detection. Therefore, selecting TBAM-YOLOv8-EMSFFN as the target detection algorithm for identifying insulator defects is well-justified.

In addition, the improved TBAM-YOLOv8-EMSFFN model has enhanced generalization ability for defect targets and has lower hardware requirements during network operation. It can be applied to insulator defect detection tasks with higher positioning accuracy requirements.

To confirm the advanced features of the TBAM-YOLOv8-EMSFFN model, a comparison was made with the latest lightweight object detection models. This includes models such as YOLOv10-N, which represent the development trend of related fields in terms of real-time performance, accuracy, and design dimensions in recent years.

All comparison models were trained and tested under the same dataset partition, with an input resolution of 640 × 640 and the same training strategy. The evaluation metrics include mAP, parameter quantity, and GFLOPs. Each experiment will be repeated five times, and the mean and standard deviation will be extracted from it. The comparison results with the latest lightweight object detection model are shown in Table 5.

**Table 5:** Comparison results with the latest lightweight object detection models.

Model	mAP (%)	Parameter quantity (M)	GFLOPs (G)
YOLOv10-N	97.2 ± 0.18	4.45	18.1
YOLOv11-N	97.5 ± 0.15	4.32	17.9
RT-DETR-R18	96.8 ± 0.22	6.21	22.4
TBAM-YOLOv8-EMSFFN	98.7 ± 0.12	4.12	17.7

As can be seen from Table 3, the model proposed in this paper is about 1.5% and 1.2% higher than that of YOLOv10-N and YOLOv11-N, respectively, and its parameters and calculation amount are at the lowest level. Compared with RT-DETR-R18, the accuracy of the model is improved by about 1.9%, and the parameters and calculation amount are reduced by 33.7% and 20.9% respectively. It can be seen that TBAM-YOLOv8-EMSFFN has a better balance between lightweight and detection accuracy, and it is a more suitable choice for the edge deployment of unmanned aerial vehicles with limited resources.

To verify the rationality that the average accuracy and the average accuracy mean value are on the high side, two additional test schemes are added. The first type is the test set without lines. All images in this set of test sets correspond to transmission lines that have not appeared in the training process samples. The second type is the cross dataset test set, which directly uses the original image in the independent insulator data set without fine-tuning the model. Neither of these two types of test sets will use the model training link, nor participate in the super parameter selection or early stop process. The corresponding results are summarized in Table 6.

**Table 6:** Independent test and cross dataset test results.

Test scheme	Average precision (%)	Average precision mean(%)	Recall rate (%)	F1 value(%)
Identically distributed test set	99.2	98.7	98.8	98.9
Test set without line	98.5	97.8	97.6	98.0
Cross dataset test set	97.6	96.5	96.2	96.9

In the test set which is consistent with the distribution of training samples, the average accuracy of the model is 99.2%, the average accuracy is 98.7%, the recall rate is 98.8%, and the F1 value is 98.9%. Using the test set composed of line data that has not appeared before, the average accuracy is 98.5%, the average accuracy is 97.8%, the recall rate is 97.6%, and the F1 value is 98.0%. The values obtained from the above groups of tests prove that the accuracy of the output results of the model is still maintained in a high range when encountering independent line scenarios and cross dataset scenarios. All high-precision test results are not caused by data leakage.

### 4.7. Performance analysis of various defects detection

Table 7 lists the average precision and recall values for all defect categories. The average precision for the normal insulator category is 99.5% and the recall rate is 99.3%, ranking first among all categories. The average precision value for the self-explosion defect is 99.3% and the recall rate is 99.0%. The identification of damaged defects and flashover defects is more difficult. The target size of these defects is smaller and they are often interfered by the complex background around them. The average precision for damaged defects and flashover defects is 98.9% and 98.8% respectively, and the recall rates are 98.5% and 98.3% respectively. After embedding the TBAM module and the EMSFFN module into the model, the recall rates for damaged defects and flashover defects are 98.5% and 98.3% respectively.

**Table 7:** Performance by category.

Category	AP(%)	Recall(%)
Normal insulator	99.5	99.3
Self‑explosion defect	99.3	99.0
Breakage defect	98.9	98.5
Flashover defect	98.8	98.3
Overall	99.2	98.8

### 4.8. Detection analysis for objects of different scales

To confirm the improvement effect of EMSFFN on the detection capability of small target defects, an analysis was conducted on the model detection performance under different target scales. The small targets, medium targets, and large targets in Table 8 were classified according to the general target detection standards. The baseline model was the YOLOv8-EMSFFN version without the small target prediction head, and the final model corresponded to TBAM-YOLOv8-EMSFFN.

**Table 8:** Comparison of detection performance under different target scales.

Object scale	AP of baseline model (%)	AP of final model (%)	Recall of baseline model (%)	Recall of final model (%)
Small object	93.5	98.4	92.8	98.0
Medium object	95.2	99.1	94.6	98.8
Large object	96.1	99.4	95.5	99.2

The statistical data in Table 8 showed that the average precision of the baseline model when processing small targets was 93.5%, and the recall rate was 92.8%; the final model with improvements had an average precision of 98.4% and a recall rate of 98.0% on small target tasks. For the medium target detection scenario, the average precision and recall rate of the final model reached 99.1% and 98.8% respectively. For the large target detection scenario, the corresponding values of the final model were 99.4% and 99.2%.

### 4.9. Deployment performance analysis of edge devices

In order to verify the deployment ability of the model in edge devices, NVIDIA Jetson Xavier NX is selected as the computing platform, and the FP16 semi-precision mode of TensorRT is used to accelerate reasoning. The input resolution is 640 × 640, the number of samples is 500, and the average of multiple inferences is taken as the result. See Table 9 for the comparison of accuracy, parameter quantity, calculation quantity, reasoning delay, throughput and average power consumption of each model.

**Table 9:** Comparison of deployment performance of edge devices.

Model	mAP (%)	Parameter quantity (M)	GFLOPs	Reasoning delay (ms)	Throughput (FPS)	Average power consumption (W)
YOLOv10-N	97.2	4.45	18.1	19.7	50.8	8.2
YOLOv11-N	97.5	4.32	17.9	20.0	50.0	8.4
RT-DETR-R18	96.8	6.21	22.4	39.8	25.1	12.0
TBAM-YOLOv8-EMSFFN	98.7	4.12	17.7	21.3	46.9	8.1

In the actual testing process at the edge, the reasoning delay of TBAM-YOLOv8-EMSFFN is 21.3ms, and its throughput reaches 46.9FPS, which can meet the real-time requirements of UAV inspection (≥30 FPS). Comparing it with YOLOv10-N and YOLOv11-N, the delay of this model is slightly increased (1.6ms higher than YOLOv10-N and 1.3ms higher than YOLOv11-N), but its detection accuracy is significantly improved, thus achieving a balance between accuracy and speed. Compared with RT-DETR-R18, the parameters of this model are reduced by 33.66%, the calculation is reduced by 20.98%, the reasoning delay is reduced by 46.48%, and the power consumption is also reduced by 32.5%, so it meets the needs of edge devices with limited resources. This model has reached an ideal equilibrium state in many different aspects, so it can be widely used in the field of intelligent power inspection of UAV edge.

As shown in Table 9, the average precision mean of TBAM-YOLOv8-EMSFFN is 98.7%, which is 1.5, 1.2, and 1.9 percentage points higher than the values of the three models YOLOv10-N, YOLOv11-N, and RT-DETR-R18 respectively. The parameter size of this model is 4.12M and the computational load is 17.7G. Both of these two indicators are smaller than those of the reference models. When deployed on edge devices, the inference delay is 21.3 milliseconds and the throughput rate can reach 46.9 frames per second, which meets the real-time requirements of unmanned aerial vehicle inspection (with a running speed of no less than 30 frames per second). Compared with YOLOv10-N and YOLOv11-N, its inference delay is 1.6 milliseconds and 1.3 milliseconds more respectively, and the detection accuracy has significantly improved. With RT-DETR-R18 as the reference, its parameter size decreases by 33.66%, the computational load decreases by 20.98%, the inference delay decreases by 46.48%, and the power consumption decreases by 32.5%. The proposed model in this paper achieves a good balance among the three indicators of accuracy, speed, and computational complexity.

## 5. Conclusion

To compensate for the accuracy decline caused by lightweight networks and address the difficulty in accurately identifying small-sized defect targets in complex backgrounds, this paper proposed a lightweight insulator defect detection algorithm based on TBAM and EMSFFN to achieve high-precision real-time detection on unmanned aerial vehicles. TBAM is integrated into YOLOv8-EMSFFN to suppress useless information such as complex backgrounds, making the network more focused on the defect surface. The feature fusion network was improved. An enhanced multi-scale feature fusion network was designed. Two layers of shallow feature maps with scales of 160 × 160 and 320 × 320 were introduced. A new classification prediction network was added after the 160 × 160 sized feature map to enhance the model’s perception ability for small targets of insulator defects. Images of different types of insulators and defects were collected, capturing target images from diverse backgrounds, angles, and perspectives. Experiments show that the optimized lightweight defect detection model TBAM-YOLOv8-EMSFFN achieves the highest AP and mAP compared with the other five algorithms. The number of model parameters and the amount of computation are 4.12M and 17.7G respectively, which are lower than those of the other five algorithms. Future work will focus on introducing distillation technology to further reduce the computational and storage overhead of the model while maintaining performance.
