# Supplementary material for: Multi-scale feature fusion network optimization and edge intelligent detection for transmission line insulator
Source: PLoS One. 2026 Oct 26;21(10):e0361403. doi: 10.1371/journal.pone.0361403 (PMC13704800; doi:10.1371/journal.pone.0361403)
Supplement: Supplementary file 1 [file pone.0361403.s001.docx]

Dear reviewers:

Thank you very much for your valuable comments on our paper. Your suggestion is of great guiding significance to improve the rigor, innovation and readability of this paper. We have carefully studied all the opinions and carefully revised and supplemented the manuscript. The following is an item-by-item reply to your comments:

Reviewer #1:

1. The data availability statement does not fully comply with journal requirements for open and reproducible research.

Response: The dataset has been uploaded to the public repository Zenodo, where it has been assigned a permanent DOI: [https://doi.org/10.5281/zenodo.21811717] .

All readers can access, download and use the data set through the above link for the verification, reproduction and expansion of the results of this study.

If there are obstacles in accessing the dataset or you need to obtain more relevant information, you can contact the corresponding author Chun Yang (Email: daixiangji865137@163.com ).

2. The statistical validation of the results is insufficient (no multiple runs, variance reporting, or significance testing).

Response: The results of several independent experiments have been supplemented, including the mean and standard deviation obtained from five repeated training, and the paired t test has been used to confirm that the performance difference is significant.

3. The reported performance is exceptionally high and requires stronger evidence to rule out overfitting or data leakage.

Response: Multiple data augmentation strategies were employed during the training process, with strict control over the number of model parameters and training epochs. When the validation performance no longer improved, the early shutdown mechanism came into play to stop the training and suppress overfitting. For the measures taken to prevent leakage in the dataset, we divide it based on the route and shooting time to avoid the same route or consecutive images appearing in both the training and testing sets. The stability of the model was further verified through cross validation.

4. The methodological contribution appears incremental and the novelty is not clearly emphasized.

Response: We have emphasized the elaboration of innovative points in the article, clearly highlighting the advantages of TBAM in terms of structure and computational efficiency, as well as the design features of EMSFFN, thus highlighting its differences from existing methods.

5. Benchmarking against more recent state-of-the-art models and stronger generalization experiments would strengthen the study.

Response: We have added relevant content on comparative experiments with YOLOv10-N, YOLOv11-N, and RT-DETR-R18 to verify the robustness and generality of the proposed method.

6. The manuscript would benefit from substantial language editing and improved clarity in technical descriptions.

Response: Thank you for your correction. We have conducted language polishing work on the entire article, correcting grammar and expression errors, and reorganizing content with unclear logic to further enhance the readability and professionalism of the entire article.

Reviewer #2:

One of the strengths of the paper is the clear motivation behind the research. The authors correctly identify two important challenges in insulator defect detection: the difficulty of detecting small targets and the limitations imposed by edge computing devices. The proposed model attempts to address these issues by integrating a multi-scale feature fusion strategy and an attention mechanism that enhances feature extraction. Another positive aspect of the work is the comparison with several widely used object detection models such as YOLOv7, YOLOv8, YOLOv9, DETR, and Faster R-CNN. This comparative evaluation helps demonstrate the potential improvement achieved by the proposed approach. The reported experimental results show very high detection performance and indicate that the proposed model can reduce computational complexity while maintaining high accuracy. The application of UAV-based inspection combined with edge intelligence is also a practical contribution that aligns with current trends in smart grid monitoring.

Despite these strengths, several aspects of the manuscript could be improved to enhance clarity, reproducibility, and scientific rigor.

First, the description of the proposed architecture could be expanded. While the paper introduces the TBAM and EMSFFN modules, the explanation of their internal structure and interaction with the base YOLOv8 model is somewhat limited. A more detailed architectural description and clearer diagrams would help readers better understand the design and novelty of the proposed approach. Additionally, the authors should provide more detailed mathematical formulations or algorithmic descriptions for the attention mechanism and feature fusion process.

Response: Thank you for the valuable comments provided by the reviewers. We have provided supplementary explanations in the corresponding sections of the main text, detailing the three parallel branch structures of TBAM, the specific role of Z-Pool operation, the embedding methods of modules in backbone networks and feature fusion networks, as well as the design logic of feature map dimension changes, fusion paths, and new prediction heads in EMSFFN to enhance readers' understanding of model design.

Second, the dataset description should be improved. The manuscript mentions that data augmentation was used to balance the dataset and increase minority class samples; however, the specific augmentation methods, dataset size before and after augmentation, and distribution of defect categories should be clearly reported. Providing this information would improve transparency and reproducibility. If possible, the authors should also clarify whether the dataset is publicly available or proprietary, and whether it can be accessed by other researchers.

Response: Thank you for your careful review. The methods of data augmentation and the distribution of samples before and after expansion have been detailed in Section 4.1. And it is explicitly stated that the dataset is a public dataset that other researchers can access.

Third, although the experimental results appear promising, the reported accuracy values (e.g., AP and mAP close to 99%) are extremely high and may raise questions regarding generalization. The authors should consider including additional validation experiments, such as cross-dataset evaluation or real-world testing scenarios, to demonstrate the robustness of the model. Ablation studies evaluating the individual contributions of TBAM and EMSFFN would also strengthen the methodological validation.

Response: We sincerely appreciate the valuable feedback provided on the experimental results of this article. The fact that the average precision (AP) and mean average precision (mAP) mentioned in the article are both close to 99%, although it can indicate that the model is effective in a specific test set, raises concerns about whether the model has overfitting and its generalization ability in complex scenarios. We agree with this viewpoint because such high accuracy lacks validation from multiple different dimensions, making it difficult to fully and comprehensively demonstrate the robustness of the model. In section 4.6, this article conducted a corresponding analysis on the deployment performance of edge devices; In section 4.4, ablation experiments were conducted on the structure of feature fusion networks, and the individual contributions of TBAM and EMSFFN will be evaluated in the future.

Fourth, the discussion section could be expanded to provide deeper analysis of the results. For example, the authors could discuss the trade-off between model accuracy, computational complexity, and inference speed on edge devices. Including metrics such as inference latency, energy consumption, or performance on embedded hardware would reinforce the claim that the model is suitable for edge intelligence applications.

Response: Thank you for your valuable suggestion. After Section 4.5, this article specifically added a section on "Performance Analysis of Edge Device Deployment", which supplemented the inference delay and throughput related indicators measured by the model on Jetson Xavier NX. At the same time, it conducted in-depth discussions on accuracy and other aspects to verify the applicability of the model in edge scenarios.

Finally, the manuscript would benefit from careful language editing. Some sections contain grammatical inconsistencies and unclear phrasing that may affect readability. Improving the clarity of the English language and restructuring certain paragraphs would make the manuscript easier to follow.

Response: Thank you for your correction. We have conducted language polishing work on the entire article, correcting grammar and expression errors, and reorganizing content with unclear logic to further enhance the readability and professionalism of the entire article.

In conclusion, the manuscript addresses an important problem in power system inspection and proposes a potentially useful deep learning architecture for insulator defect detection on edge devices. With improvements in methodological explanation, dataset transparency, experimental validation, and language clarity, the paper could make a valuable contribution to research in intelligent inspection systems and computer vision applications in power engineering.

Response: Sincerely thank you for your affirmation of this research work and constructive suggestions for improving this article. We have strictly followed your suggestions in this round of revisions to systematically improve the method description, data transparency, experimental verification, and language expression. We believe that these modifications significantly enhance the completeness and rigor of the paper, providing better reference value for the research on intelligent detection systems and power engineering applications. Thank you again for your valuable time and professional guidance.
