# Supplementary material for: Multi-scale feature fusion network optimization and edge intelligent detection for transmission line insulator
Source: PLoS One. 2026 Oct 26;21(10):e0361403. doi: 10.1371/journal.pone.0361403 (PMC13704800; doi:10.1371/journal.pone.0361403)
Supplement: Supplementary file 2 [file pone.0361403.s002.docx]

Dear Editor and Reviewers,

We would like to express our sincere gratitude to the editor and all reviewers for the valuable time and constructive comments on our manuscript entitled “Multi-Scale Feature Fusion Network Optimization and Edge Intelligent Detection for Transmission Line Insulator.” These comments and suggestions are greatly helpful in improving the clarity, rigor, and overall quality of our work. We have carefully considered all the comments and revised the manuscript accordingly. Below we provide a point-by-point response to each comment, and the corresponding revisions are highlighted in the revised manuscript. We sincerely hope that the revised version addresses all the concerns, and we would be glad to make further improvements if needed.

Sincerely,

Chun Yang, Yining Ma

Reviewer #1: The manuscript addresses an important problem and presents a potentially useful lightweight detection framework. However, the following issues should be addressed:

1. Please strengthen validation of the exceptionally high AP/mAP results using an independent or cross-dataset test set.

Response: Thank you for your valuable comments. We carefully read the relevant comments put forward by the reviewers, and added the line test set and cross data set test set that have not appeared before. The corresponding parameter settings and experimental results have been placed in Section 4.5. From the final test data, it can be seen that the high average precision and average precision obtained by the model do not have the problem of data leakage, nor is it the result of over fitting for a specific line and a specific data set.

2. Please clarify the data-splitting procedure and confirm that augmented versions of the same images were not distributed across different datasets.

Response: This article has clearly defined the specific operation steps for data division. First, the division process is completed, and then the enhancement operation is carried out. Only the training set will undergo enhancement processing, while the validation set and test set will retain the original image content. Multiple enhanced versions generated from the same image will not be assigned to different subsets, which can avoid the problem of data leakage and ensure the fairness of the evaluation process.

3. Please provide clearer component-wise ablation results to demonstrate the individual contributions of TBAM and EMSFFN.

Response: This update has added a component-level ablation table, which presents the actual performance of TBAM at different positions, as well as the operation effects of each component within EMSFFN. The relevant data shows that both TBAM and EMSFFN can play a clear role, and when used together, the balance between accuracy and efficiency can reach the optimal state.

4. Please clarify the statistical methodology, including the rationale for five repeated runs and the paired t-test.

Response: This article has clearly explained the statistical methods used. Five repetitions were set in the experimental process to reduce the interference caused by random factors. The applicable scenario of the paired t-test is when two models are compared under the same data partitioning rule. The relevant p-values and standard deviations have been fully attached.

5. Please discuss the trade-off between detection accuracy, inference speed, and computational complexity more carefully.

Response: This article has added a more detailed cost-benefit analysis. The proposed model runs slightly slower than YOLOv10-N and YOLOv11-N, but it has better accuracy and fully meets the requirements related to real-time performance. Compared with RT-DETR-R18, this model has significant advantages in terms of detection accuracy, inference speed, and computational complexity.

6. Please clarify the specific novelty of TBAM and EMSFFN compared with existing attention and feature-fusion methods.

Response: This article has clearly explained the innovative contents of the TBAM and EMSFFN modules. TBAM does not directly superimpose various existing attention modules. The overall structure uses a special design of three branches combined with Z-Pool. EMSFFN adds two shallow feature maps and pairs them with a dedicated prediction head specifically adapted for small targets. When the two modules are combined, they are developed to complete the task of insulator defect detection in the edge device operation scenario.

7. Please carefully proofread the manuscript to improve language, clarity, and consistency.

Response: We have carefully reviewed the entire text, correcting terms, grammar, formula formatting and table consistency. All the modifications have been marked in the revised version.

Overall, the study is promising, but these issues should be addressed before publication.

Response: Thank you for your constructive comments. We appreciate your positive assessment of this work. We have carefully addressed all the raised concerns in the revised manuscript.

Reviewer #2: A lightweight insulator defect detection algorithm based on TBAM and EMSFFN to achieve high-precision real-time detection on unmanned aerial vehicles. is proposed in this paper. The paper is well-organized and the topic is interesting. There is potential in this manuscript, but it requires further work.

1. The EDiou loss function is proposed in the article. It is suggested to further supplement with a comparison with the original CIou to clarify its actual improvement on localization performance.

Response: This paper has conducted a comparative experiment between CIoU and EDIoU. The results show that EDIoU can optimize the positioning performance and performs better in adapting to small target defects.

2. The article mentions the use of 5-fold cross-validation and unseen line image verification to assess generalization ability, but the relevant experimental settings and result presentation are still not clear enough. It is recommended to supplement with specific verification methods and main results.

Response: This paper has completed the relevant parameter settings and core test results of five-fold cross-validation and unseen-line validation. When dividing the data set, the line number is used as the grouping basis, and all images collected on the same line will not be included in the training set and the test set at the same time.

3. The current model has a relatively high overall mAP. It is recommended to further clarify the AP and Recall for each defect category to analyze the specific detection performance of categories such as damage and flashover.

Response: In this paper, the average precision and recall rate have been counted by category. It can be seen from the comparative data that the detection performance of the proposed model is optimized for the categories with the highest recognition difficulty, such as damage and flashover.

4. In Figure 6, the detection performance decreases after the addition of CBAM. It is recommended to further analyze the reasons for the performance differences caused by different attention mechanisms, in conjunction with feature response visualization or typical detection results.

Response: This article has finished sorting out the reasons for the performance decline of CBAM. This reason corresponds to the attention mechanism arranged in order of channel and space, which limits the weak characteristic response of small targets in the shallow network.

5. EMSFFN is primarily designed for small target defects. It is recommended to further analyze its detection performance under different target scales to verify its improvement in small target detection capability.

Response: Thank you for the suggestion. This paper has supplemented the content of detection performance analysis corresponding to different target scales. The average accuracy and recall rate of the final model for small targets are 98.4% and 98.0%, respectively, which are 4.9% and 5.2% higher than the benchmark model, and the corresponding improvement range is higher than the relevant values of the medium target and the large target. The enhancement effect of EMSFFN on the defect detection ability of small targets really exists.

Reviewer #3: The manuscript is suitable for publication in its current form.

I have no further comments or concerns. The work is complete and clearly presented, and I recommend acceptance.

Response: We sincerely thank the reviewer for their positive evaluation.
