# Supplementary material for: Multi-scale feature fusion network optimization and edge intelligent detection for transmission line insulator
Source: PLoS One. 2026 Oct 26;21(10):e0361403. doi: 10.1371/journal.pone.0361403 (PMC13704800; doi:10.1371/journal.pone.0361403)
Supplement: Peer review history

# Author comment: Author response to Decision Letter 0

**DOI:** 10.1371/journal.pone.0361403.r001

6 Aug 2026

Attachment Submitted filename: Response to Reviewers.docx

# Peer review: Decision Letter 0

**DOI:** 10.1371/journal.pone.0361403.r002

**Authors:** Guangyu Sun

3 Sep 2026

Dear Dr. Yang,

Thank you for submitting your manuscript to PLOS One. After careful consideration, we feel that it has merit but does not fully meet PLOS One’s publication criteria as it currently stands. Therefore, we invite you to submit a revised version of the manuscript that addresses the points raised during the review process.

Please submit your revised manuscript by Nov 02 2026 11:59PM. If you will need more time than this to complete your revisions, please reply to this message or contact the journal office at plosone@plos.org. When you're ready to submit your revision, log on to https://www.editorialmanager.com/pone/ and select the 'Submissions Needing Revision' folder to locate your manuscript file.

- A letter that responds to each point raised by the academic editor and reviewer(s). You should upload this letter as a separate file labeled 'Response to Reviewers'.
- A marked-up copy of your manuscript that highlights changes made to the original version. You should upload this as a separate file labeled 'Revised Manuscript with Track Changes'.
- An unmarked version of your revised paper without tracked changes. You should upload this as a separate file labeled 'Manuscript'.

If you would like to make changes to your financial disclosure, please include your updated statement in your cover letter. Guidelines for resubmitting your figure files are available below the reviewer comments at the end of this letter.

If applicable, we recommend that you deposit your laboratory protocols in protocols.io to enhance the reproducibility of your results. Protocols.io assigns your protocol its own identifier (DOI) so that it can be cited independently in the future. For instructions see: https://journals.plos.org/plosone/s/submission-guidelines#loc-laboratory-protocols. Additionally, PLOS One offers an option for publishing peer-reviewed Lab Protocol articles, which describe protocols hosted on protocols.io. Read more information on sharing protocols at https://plos.org/protocols?utm_medium=editorial-email&utm_source=authorletters&utm_campaign=protocols.

As the corresponding author, your ORCID iD is verified in the submission system and will appear in the published article. PLOS supports the use of ORCID, and we encourage all coauthors to register for an ORCID iD and use it as well. Please encourage your coauthors to verify their ORCID iD within the submission system before final acceptance, as unverified ORCID iDs will not appear in the published article. *Only*  the individual author can complete the verification step; PLOS staff *cannot*  verify ORCID iDs on behalf of authors.

We look forward to receiving your revised manuscript.

Kind regards,

Guangyu Sun, Ph.D

Academic Editor

PLOS One

Journal Requirements:

When submitting your revision, we need you to address these additional requirements.

1.Please ensure that your manuscript meets PLOS ONE’s style requirements, including those for file naming. The PLOS ONE style templates can be found at

https://journals.plos.org/plosone/s/file?id=wjVg/PLOSOne_formatting_sample_main_body.pdf and

https://journals.plos.org/plosone/s/file?id=ba62/PLOSOne_formatting_sample_title_authors_affiliations.pdf

2. Please note that PLOS One has specific guidelines on code sharing for submissions in which author-generated code underpins the findings in the manuscript. In these cases, we expect all author-generated code to be made available without restrictions upon publication of the work. Please review our guidelines at https://journals.plos.org/plosone/s/materials-and-software-sharing#loc-sharing-code and ensure that your code is shared in a way that follows best practice and facilitates reproducibility and reuse.

3. Thank you for stating the following in your manuscript:

“This study was supported by the Energy Development Research Institute of China Southern Power Grid Co., LTD., where the author is located, including the provision of research materials such as insulator image datasets and author compensation support. The funder did not exert influence in research design, data processing and analysis, result interpretation, paper writing, and publication decisions, but as the author’s affiliation, they made significant contributions in data collection and research materials. The specific roles of the authors in the research have been clearly stated in the ‘author contributions’ section.”

We note that you have provided funding information that is not currently declared in your Funding Statement. However, funding information should not appear in the Acknowledgments section or other areas of your manuscript. We will only publish funding information present in the Funding Statement section of the online submission form.

Please remove any funding-related text from the manuscript and let us know how you would like to update your Funding Statement. Currently, your Funding Statement reads as follows:

“The author(s) received no specific funding for this work.”

Please include your amended statements within your cover letter; we will change the online submission form on your behalf.

4. Thank you for stating the following in the Competing Interests section:

“The authors have declared that no competing interests exist.”

We note that one or more of the authors are employed by a commercial company: China Southern Power Grid Co Ltd

1.        Please provide an amended Funding Statement declaring this commercial affiliation, as well as a statement regarding the Role of Funders in your study. If the funding organization did not play a role in the study design, data collection and analysis, decision to publish, or preparation of the manuscript and only provided financial support in the form of authors' salaries and/or research materials, please review your statements relating to the author contributions, and ensure you have specifically and accurately indicated the role(s) that these authors had in your study. You can update author roles in the Author Contributions section of the online submission form.

Please also include the following statement within your amended Funding Statement.

“The funder provided support in the form of salaries for authors [insert relevant initials], but did not have any additional role in the study design, data collection and analysis, decision to publish, or preparation of the manuscript. The specific roles of these authors are articulated in the ‘author contributions’ section.”

If your commercial affiliation did play a role in your study, please state and explain this role within your updated Funding Statement.

2. Please also provide an updated Competing Interests Statement declaring this commercial affiliation along with any other relevant declarations relating to employment, consultancy, patents, products in development, or marketed products, etc.

Within your Competing Interests Statement, please confirm that this commercial affiliation does not alter your adherence to all PLOS ONE policies on sharing data and materials by including the following statement: "This does not alter our adherence to  PLOS ONE policies on sharing data and materials.” (as detailed online in our guide for authors http://journals.plos.org/plosone/s/competing-interests) . If this adherence statement is not accurate and  there are restrictions on sharing of data and/or materials, please state these. Please note that we cannot proceed with consideration of your article until this information has been declared.

Please include both an updated Funding Statement and Competing Interests Statement in your cover letter. We will change the online submission form on your behalf.

5. Thank you for stating the following in the Competing Interests section:

“The authors have declared that no competing interests exist.”

We note that you received funding from a commercial source: Energy Development Research Institute of China Southern Power Grid Co., LTD.

Please provide an amended Competing Interests Statement that explicitly states this commercial funder, along with any other relevant declarations relating to employment, consultancy, patents, products in development, marketed products, etc.

Within this Competing Interests Statement, please confirm that this does not alter your adherence to all PLOS ONE policies on sharing data and materials by including the following statement: "This does not alter our adherence to PLOS ONE policies on sharing data and materials.” (as detailed online in our guide for authors http://journals.plos.org/plosone/s/competing-interests).  If there are restrictions on sharing of data and/or materials, please state these. Please note that we cannot proceed with consideration of your article until this information has been declared.

Please include your amended Competing Interests Statement within your cover letter. We will change the online submission form on your behalf.

6. If the reviewer comments include a recommendation to cite specific previously published works, please review and evaluate these publications to determine whether they are relevant and should be cited. There is no requirement to cite these works unless the editor has indicated otherwise.

[Note: HTML markup is below. Please do not edit.]

Reviewers' comments:

Reviewer’s Responses to Questions

**Comments to the Author**

1. Is the manuscript technically sound, and do the data support the conclusions?

Reviewer #1: No

Reviewer #2: Yes

Reviewer #3: Yes

**********

2. Has the statistical analysis been performed appropriately and rigorously?

Reviewer #1: No

Reviewer #2: Yes

Reviewer #3: Yes

**********

3. Have the authors made all data underlying the findings in their manuscript fully available?

The PLOS Data policy

Reviewer #1: Yes

Reviewer #2: Yes

Reviewer #3: Yes

**********

4. Is the manuscript presented in an intelligible fashion and written in standard English?

Reviewer #1: Yes

Reviewer #2: Yes

Reviewer #3: Yes

**********

Reviewer #1: The manuscript addresses an important problem and presents a potentially useful lightweight detection framework. However, the following issues should be addressed:

1. Please strengthen validation of the exceptionally high AP/mAP results using an independent or cross-dataset test set.

2. Please clarify the data-splitting procedure and confirm that augmented versions of the same images were not distributed across different datasets.

3. Please provide clearer component-wise ablation results to demonstrate the individual contributions of TBAM and EMSFFN.

4. Please clarify the statistical methodology, including the rationale for five repeated runs and the paired t-test.

5. Please discuss the trade-off between detection accuracy, inference speed, and computational complexity more carefully.

6. Please clarify the specific novelty of TBAM and EMSFFN compared with existing attention and feature-fusion methods.

7. Please carefully proofread the manuscript to improve language, clarity, and consistency.

Overall, the study is promising, but these issues should be addressed before publication.

Reviewer #2: A lightweight insulator defect detection algorithm based on TBAM and EMSFFN to achieve high-precision real-time detection on unmanned aerial vehicles. is proposed in this paper. The paper is well-organized and the topic is interesting. There is potential in this manuscript, but it requires further work.

1. The EDiou loss function is proposed in the article. It is suggested to further supplement with a comparison with the original CIou to clarify its actual improvement on localization performance.

2. The article mentions the use of 5-fold cross-validation and unseen line image verification to assess generalization ability, but the relevant experimental settings and result presentation are still not clear enough. It is recommended to supplement with specific verification methods and main results.

3. The current model has a relatively high overall mAP. It is recommended to further clarify the AP and Recall for each defect category to analyze the specific detection performance of categories such as damage and flashover.

4. In Figure 6, the detection performance decreases after the addition of CBAM. It is recommended to further analyze the reasons for the performance differences caused by different attention mechanisms, in conjunction with feature response visualization or typical detection results.

5. EMSFFN is primarily designed for small target defects. It is recommended to further analyze its detection performance under different target scales to verify its improvement in small target detection capability.

Reviewer #3: The manuscript is suitable for publication in its current form.

I have no further comments or concerns. The work is complete and clearly presented, and I recommend acceptance.

**********

what does this mean?). If published, this will include your full peer review and any attached files.

If you choose “no”, your identity will remain anonymous but your review may still be made public.

**Do you want your identity to be public for this peer review?**  For information about this choice, including consent withdrawal, please see our Privacy Policy

Reviewer #1: No

Reviewer #2: No

Reviewer #3: **Yes:**  Elaheh Yaghoubi

**********

[NOTE: If reviewer comments were submitted as an attachment file, they will be attached to this email and accessible via the submission site. Please log into your account, locate the manuscript record, and check for the action link "View Attachments". If this link does not appear, there are no attachment files.]

To ensure your figures meet our technical requirements, please review our figure guidelines: https://journals.plos.org/plosone/s/figures

You may also use PLOS’s free figure tool, NAAS, to help you prepare publication quality figures: https://journals.plos.org/plosone/s/figures#loc-tools-for-figure-preparation.

NAAS will assess whether your figures meet our technical requirements by comparing each figure against our figure specifications.

# Author comment: Author response to Decision Letter 1

**DOI:** 10.1371/journal.pone.0361403.r003

27 Sep 2026

Reviewer #1: The manuscript addresses an important problem and presents a potentially useful lightweight detection framework. However, the following issues should be addressed:

1. Please strengthen validation of the exceptionally high AP/mAP results using an independent or cross-dataset test set.

Response: Thank you for your valuable comments. We carefully read the relevant comments put forward by the reviewers, and added the line test set and cross data set test set that have not appeared before. The corresponding parameter settings and experimental results have been placed in Section 4.5. From the final test data, it can be seen that the high average precision and average precision obtained by the model do not have the problem of data leakage, nor is it the result of over fitting for a specific line and a specific data set.

2. Please clarify the data-splitting procedure and confirm that augmented versions of the same images were not distributed across different datasets.

Response: This article has clearly defined the specific operation steps for data division. First, the division process is completed, and then the enhancement operation is carried out. Only the training set will undergo enhancement processing, while the validation set and test set will retain the original image content. Multiple enhanced versions generated from the same image will not be assigned to different subsets, which can avoid the problem of data leakage and ensure the fairness of the evaluation process.

3. Please provide clearer component-wise ablation results to demonstrate the individual contributions of TBAM and EMSFFN.

Response: This update has added a component-level ablation table, which presents the actual performance of TBAM at different positions, as well as the operation effects of each component within EMSFFN. The relevant data shows that both TBAM and EMSFFN can play a clear role, and when used together, the balance between accuracy and efficiency can reach the optimal state.

4. Please clarify the statistical methodology, including the rationale for five repeated runs and the paired t-test.

Response: This article has clearly explained the statistical methods used. Five repetitions were set in the experimental process to reduce the interference caused by random factors. The applicable scenario of the paired t-test is when two models are compared under the same data partitioning rule. The relevant p-values and standard deviations have been fully attached.

5. Please discuss the trade-off between detection accuracy, inference speed, and computational complexity more carefully.

Response: This article has added a more detailed cost-benefit analysis. The proposed model runs slightly slower than YOLOv10-N and YOLOv11-N, but it has better accuracy and fully meets the requirements related to real-time performance. Compared with RT-DETR-R18, this model has significant advantages in terms of detection accuracy, inference speed, and computational complexity.

6.Please clarify the specific novelty of TBAM and EMSFFN compared with existing attention and feature-fusion methods.

Response: This article has clearly explained the innovative contents of the TBAM and EMSFFN modules. TBAM does not directly superimpose various existing attention modules. The overall structure uses a special design of three branches combined with Z-Pool. EMSFFN adds two shallow feature maps and pairs them with a dedicated prediction head specifically adapted for small targets. When the two modules are combined, they are developed to complete the task of insulator defect detection in the edge device operation scenario.

7. Please carefully proofread the manuscript to improve language, clarity, and consistency.

Response: We have carefully reviewed the entire text, correcting terms, grammar, formula formatting and table consistency. All the modifications have been marked in the revised version.

Overall, the study is promising, but these issues should be addressed before publication.

Response: Thank you for your constructive comments. We appreciate your positive assessment of this work. We have carefully addressed all the raised concerns in the revised manuscript.

Reviewer #2: A lightweight insulator defect detection algorithm based on TBAM and EMSFFN to achieve high-precision real-time detection on unmanned aerial vehicles. is proposed in this paper. The paper is well-organized and the topic is interesting. There is potential in this manuscript, but it requires further work.

1. The EDiou loss function is proposed in the article. It is suggested to further supplement with a comparison with the original CIou to clarify its actual improvement on localization performance.

Response: This paper has conducted a comparative experiment between CIoU and EDIoU. The results show that EDIoU can optimize the positioning performance and performs better in adapting to small target defects.

2. The article mentions the use of 5-fold cross-validation and unseen line image verification to assess generalization ability, but the relevant experimental settings and result presentation are still not clear enough. It is recommended to supplement with specific verification methods and main results.

Response: This paper has completed the relevant parameter settings and core test results of five-fold cross-validation and unseen-line validation. When dividing the data set, the line number is used as the grouping basis, and all images collected on the same line will not be included in the training set and the test set at the same time.

3. The current model has a relatively high overall mAP. It is recommended to further clarify the AP and Recall for each defect category to analyze the specific detection performance of categories such as damage and flashover.

Response: In this paper, the average precision and recall rate have been counted by category. It can be seen from the comparative data that the detection performance of the proposed model is optimized for the categories with the highest recognition difficulty, such as damage and flashover.

4. In Figure 6, the detection performance decreases after the addition of CBAM. It is recommended to further analyze the reasons for the performance differences caused by different attention mechanisms, in conjunction with feature response visualization or typical detection results.

Response: This article has finished sorting out the reasons for the performance decline of CBAM. This reason corresponds to the attention mechanism arranged in order of channel and space, which limits the weak characteristic response of small targets in the shallow network.

5. EMSFFN is primarily designed for small target defects. It is recommended to further analyze its detection performance under different target scales to verify its improvement in small target detection capability.

Response: Thank you for the suggestion. This paper has supplemented the content of detection performance analysis corresponding to different target scales. The average accuracy and recall rate of the final model for small targets are 98.4% and 98.0%, respectively, which are 4.9% and 5.2% higher than the benchmark model, and the corresponding improvement range is higher than the relevant values of the medium target and the large target. The enhancement effect of EMSFFN on the defect detection ability of small targets really exists.

Reviewer #3: The manuscript is suitable for publication in its current form.

I have no further comments or concerns. The work is complete and clearly presented, and I recommend acceptance.

Response: We sincerely thank the reviewer for their positive evaluation.

Attachment Submitted filename: Response_to_Reviewers_auresp_1.docx

# Peer review: Decision Letter 1

**DOI:** 10.1371/journal.pone.0361403.r004

**Authors:** Guangyu Sun

12 Oct 2026

Multi-Scale Feature Fusion Network Optimization and Edge Intelligent Detection for Transmission Line Insulator

PONE-D-26-39386R1

Dear Dr. Yang,

We’re pleased to inform you that your manuscript has been judged scientifically suitable for publication and will be formally accepted for publication once it meets all outstanding technical requirements.

Within one week, you’ll receive an e-mail detailing the required amendments. When these have been addressed, you’ll receive a formal acceptance letter and your manuscript will be scheduled for publication.

An invoice will be generated when your article is formally accepted. Please note, if your institution has a publishing partnership with PLOS and your article meets the relevant criteria, all or part of your publication costs will be covered. Please make sure your user information is up-to-date by logging into Editorial Manager at Editorial Manager® and clicking the ‘Update My Information' link at the top of the page. For questions related to billing, please contact billing support.

If your institution or institutions have a press office, please notify them about your upcoming paper to help maximize its impact. If they’ll be preparing press materials, please inform our press team as soon as possible -- no later than 48 hours after receiving the formal acceptance. Your manuscript will remain under strict press embargo until 2 pm Eastern Time on the date of publication. For more information, please contact onepress@plos.org.

Kind regards,

Guangyu Sun, Ph.D

Academic Editor

PLOS One

Additional Editor Comments (optional):

Reviewers' comments:

Reviewer’s Responses to Questions

**Comments to the Author**

Reviewer #2: (No Response)

**********

2. Is the manuscript technically sound, and do the data support the conclusions?

Reviewer #2: (No Response)

**********

3. Has the statistical analysis been performed appropriately and rigorously?

Reviewer #2: (No Response)

**********

4. Have the authors made all data underlying the findings in their manuscript fully available?

The PLOS Data policy

Reviewer #2: (No Response)

**********

5. Is the manuscript presented in an intelligible fashion and written in standard English?

Reviewer #2: (No Response)

**********

Reviewer #2: The author has already made a good supplement, and I have no further questions. The author has already made a good supplement, and I have no further questions.

**********

what does this mean?). If published, this will include your full peer review and any attached files.

If you choose “no”, your identity will remain anonymous but your review may still be made public.

**Do you want your identity to be public for this peer review?**  For information about this choice, including consent withdrawal, please see our Privacy Policy

Reviewer #2: No

**********

# Editor report: Acceptance letter

**DOI:** 10.1371/journal.pone.0361403.r005

**Authors:** Guangyu Sun

PONE-D-26-39386R1

PLOS One

Dear Dr. Yang,

I'm pleased to inform you that your manuscript has been deemed suitable for publication in PLOS One. Congratulations! Your manuscript is now being handed over to our production team.

At this stage, our production department will prepare your paper for publication. This includes ensuring the following:

* All references, tables, and figures are properly cited

* All relevant supporting information is included in the manuscript submission,

* There are no issues that prevent the paper from being properly typeset

You will receive further instructions from the production team, including instructions on how to review your proof when it is ready. Please keep in mind that we are working through a large volume of accepted articles, so please give us a few days to review your paper and let you know the next and final steps.

Lastly, if your institution or institutions have a press office, please let them know about your upcoming paper now to help maximize its impact. If they'll be preparing press materials, please inform our press team within the next 48 hours. Your manuscript will remain under strict press embargo until 2 pm Eastern Time on the date of publication. For more information, please contact onepress@plos.org.

You will receive an invoice from PLOS for your publication fee after your manuscript has reached the completed accept phase. If you receive an email requesting payment before acceptance or for any other service, this may be a phishing scheme. Learn how to identify phishing emails and protect your accounts at https://explore.plos.org/phishing.

If we can help with anything else, please email us at customercare@plos.org.

Thank you for submitting your work to PLOS One and supporting open access.

Kind regards,

PLOS One Editorial Office Staff

on behalf of

Dr. Guangyu Sun

Academic Editor

PLOS One
